# Supplementary material for: Association between socioeconomic status and pain, function and pain catastrophizing at presentation for total knee arthroplasty
Source: BMC Musculoskelet Disord. 2015 Feb 7;16:18. doi: 10.1186/s12891-015-0475-8 (PMC4329215; doi:10.1186/s12891-015-0475-8)
Supplement: Additional file 1: Table S1. — Unadjusted and adjusted models indicating the percentage and 95% CI of subjects with high pain (WOMAC >55), low function (WOMAC >55) or high pain catastrophizing (PCS ≥16) stratified by individual-level SES. [file 12891_2015_475_MOESM1_ESM.docx]

**Association between Socioeconomic Status and Pain, Function and Pain Catastrophizing at Presentation for Total Knee Arthroplasty**

Candace H. Feldman (1)

Yan Dong (2)

Jeffrey N. Katz (3)

Laurel A. Donnell-Fink (4)

Elena Losina (5)

1. Corresponding Author. Brigham and Women’s Hospital, Division of Rheumatology, Immunology and Allergy; Harvard School of Public Health, 75 Francis Street, Boston, MA 02115. Phone: 617-732-5325; Fax: 617-525-1010. Email: [chfeldman@partners.org](mailto:chfeldman@partners.org)
2. Brigham and Women’s Hospital, Orthopedics and Arthritis Center for Outcome Research (OrACORe), 75 Francis Street, Boston, MA 02115. [yad509@mail.harvard.edu](mailto:yad509@mail.harvard.edu)
3. Brigham and Women’s Hospital, Division of Rheumatology, Immunology and Allergy; Brigham and Women’s Hospital, Orthopedics and Arthritis Center for Outcomes Research; Harvard School of Public Health, 75 Francis Street, Boston, MA 02115. [jnkatz@partners.org](mailto:jnkatz@partners.org)
4. Brigham and Women’s Hospital, Orthopedics and Arthritis Center for Outcome Research (OrACORe), 75 Francis Street, Boston, MA 02115. [Ldonnell-fink@partners.org](mailto:Ldonnell-fink@partners.org)
5. Brigham and Women’s Hospital, Orthopedics and Arthritis Center for Outcome Research (OrACORe), 75 Francis Street, Boston, MA 02115. [elosina@partners.org](mailto:elosina@partners.org)

**Abstract**

**Background:** Patients with higher socioeconomic status (SES) are shown to have better total knee arthroplasty (TKA) outcomes compared to those with lower SES. The relationship between SES and factors that influence TKA use and outcomes is understudied. We examined the association between SES and pain, function and pain catastrophizing at presentation for TKA.

**Methods:** In patients undergoing TKA at an academic center, we obtained preoperative pain and functional status (WOMAC Index 0-100, 100 worst), pain catastrophizing (PCS, >16 high), and mental health (MHI-5, <68 poor). We described individual-level SES using education as a proxy, and area-level SES using a validated composite index linking geocoded addresses to U.S. Census data. We measured associations between these indicators and pain, function and pain catastrophizing, adjusting for age, sex and BMI.

**Results.**  Among 316 patients, mean age was 65.9 (SD 8.7), 59% were female, and 88% were Caucasian; 17% achieved less than college education and 62% were college graduates. The median area SES index score was 59 (U.S. median 51). Bivariate analyses demonstrated associations between higher individual- and area-level SES and lower pain, higher function and less pain catastrophizing (all p<0.05). Adjusted analyses demonstrated statistically significant associations between higher individual- and area-level SES and better function and less pain.

**Conclusion.** In this cohort, patients with higher individual- and area-level SES had lower pain and higher function at the time of TKA than lower SES patients. Further research is needed to assess what constitutes appropriate levels of pain and function to undergo TKA in these higher SES groups.

Keywords: Total Knee arthroplasty, Socioeconomic status, Pain, Osteoarthritis

**Background**

In the U.S. population, rates of total knee and hip joint arthroplasty continue to rise with persistent racial, ethnic and geographic disparities in procedure use and outcomes [[1-4](#_ENREF_1)]. Studies to date have examined a number of factors relating to socioeconomic status (SES) and joint arthroplasty including differences in access, need, willingness to undergo the procedure, and outcomes. Prior studies based in Europe and Australia examined the influence of SES on joint arthroplasty and demonstrated that the lowest income individuals and those from the most deprived areas presented for surgery with the poorest health-related quality of life, had the lowest **r**ates of surgery, and experienced more post-operative adverse events than higher income individuals and those from less deprived areas [[5-8](#_ENREF_5)]. A Canadian-based study surveyed individuals with moderate-to-severe knee and hip osteoarthritis and found that less education and lower income were associated with increased need for arthroplasty (based on Western Ontario and McMaster Universities Arthritis Index (WOMAC) score >39 without a contraindication to surgery), and that these lower SES individuals were equally willing to undergo the procedure as their higher SES counterparts [[9](#_ENREF_9)]. In the UK, a review of hospitalizations for primary and revision hip and knee replacements between 1991 and 2001 demonstrated that the most deprived fifth of the population experienced significantly lower incidence rates of surgery.[[5](#_ENREF_5)] In each of these studies, lower SES was shown to be associated with reduced arthroplasty use or with adverse outcomes.

Prior studies have also investigated the relationship between race/ethnicity and utilization of joint arthroplasty.[[10-12](#_ENREF_10)] However, while a subset of these studies adjusted for SES-related variables, the specific association between SES and key factors shown to influence surgical outcomes- pain, function and pain catastrophizing were not examined.[[10](#_ENREF_10), [13](#_ENREF_13)] One study based in New Zealand found that individuals from lower social classes experienced a greater increase in knee pain and disability over a seven-year period, compared to those from higher social classes.[[14](#_ENREF_14)] A recent study demonstrated associations between increased pain and disability among lower SES individuals with radiographic knee osteoarthritis. However, this study included all individuals with radiographic evidence of knee osteoarthritis, and was not specifically focused on the subset of the population with severe enough disease to present for arthroplasty.[[15](#_ENREF_15)]

Increased pain and functional limitations at presentation for arthroplasty are predictive of poorer postoperative outcomes [[16-18](#_ENREF_16)]. Heightened pain catastrophizing also contributes to pain at presentation, pain-related disability, psychological distress, and postoperative outcomes [[19-22](#_ENREF_19)]. While studies demonstrate a potential relationship between SES and pain and with arthroplasty utilization and outcomes, an understanding of the association between SES and these critical factors specifically at the time of presentation might allow for the development of interventions to improve access and reduce disparities in care.

We therefore aimed to investigate the association of SES, both at the individual and area levels, with preoperative pain, function and pain catastrophizing.[[23](#_ENREF_23)] We hypothesized that a gradient may exist whereby greater SES would be associated with lower levels of pain and better function at presentation for TKA, possibly as a result of earlier access to orthopedic care.

**Methods**

Patient Population

We obtained baseline data from a survey administered to patients at a preoperative assessment visit prior to TKA, at which time they were enrolled in the Adding Value in Knee Arthroplasty (AViKA) Observational Cohort or the AViKA Care Navigator Intervention Study, between 2010 and 2013. All of the patients were scheduled for TKA at one urban, academic medical center. Demographic information including age, sex, race/ethnicity, home address, body mass index (BMI), and smoking status were obtained by self-report. The studies were approved by the Partners Human Research Committee (2010P-001135, 2010P-002597). The AViKA Navigator trial was registered at ClinicalTrials.gov (NCT01540851).

Individual Level SES: Educational Attainment

Educational attainment, a frequently used proxy for individual-level SES [[23](#_ENREF_23)], was obtained by self-report in response to the question: “What is the highest level of education you achieved?” and included the categories of 1) did not graduate from high school, 2) graduated from high school but did not attend college or technical school, 3) graduated from high school and attended college or technical school and 4) graduated from high school and graduated from college or technical school. Given the small number of individuals indicating less than high school education in our cohort, the first and the second groups were combined and categorized as “less than college.” This question of highest level of education achieved, with the aforementioned categories, is widely used in U.S.-based studies of adults as a relatively static, well-demarcated measure across the U.S population of individual-level SES.

Area-Level SES: Composite Index

Area-level composite measures, typically derived by geocoding addresses that link to U.S. Census variables, are often utilized when individual measures are unavailable, and may capture neighborhood factors such as social cohesion, social capital, and neighborhood safety, that contribute significantly and often independently to the health of individuals [[24-26](#_ENREF_24)].

We used the Geographic Information System (GIS) to geocode individual home addresses. Addresses of the nearest post office were used if a Post Office Box was provided. Federal Information Processing Standard (FIPS) codes were obtained for each address and were linked to U.S. Census and American Community Survey data at the block group level. The block group is the smallest geographic unit for which the variables needed are published and includes populations of 600 to 3000 people. We then utilized a composite measure for SES that was validated in the U.S. Medicare population [[27](#_ENREF_27)]. The SES variables included occupation (percentage of persons >16 in the labor force who are unemployed and actively seeking work), income (percentage of persons below the federally defined poverty line and median household income), wealth (median value of owner-occupied homes), education (percentage of persons aged >25 with less than a 12^th^ grade education and percentage of persons aged >25 years with at least four years of college) and crowding (percentage of households containing >1 person per room). Individual measures were weighted using values previously determined through principal components analysis as defined by the original index validated in the Medicare population [[27](#_ENREF_27)]. SES index scores were standardized (0-100) and divided into quartiles (<55 (lowest), 56-59, 60-63 and >63 (highest)).

Mental Health Status Assessment

We used the Mental Health Index-5 (MHI-5) as a measure of general mental health. The MHI-5 has been validated to assess mental health status and as a screening for mood disorders[[28](#_ENREF_28), [29](#_ENREF_29)]. It includes five questions that are summed and scaled from 0 to 100 using a linear transformation. Higher scores (>68 in our cohort) are indicative of better mental health and lower scores, <68, of poorer mental health. This cutoff was used in a previous study with >68 representing mild, moderate and severe depressive symptoms on the MHI-5 survey.[[30](#_ENREF_30)]

Outcomes: Pain, Function and Pain Catastrophizing Assessment

To obtain baseline pain and function, we used the WOMAC Osteoarthritis index [[31](#_ENREF_31)]. This is a validated and widely used measure of lower extremity pain and functional status in patients with osteoarthritis. Responses were summed and scaled into 0 to 100 using linear transformation separately for pain and for function. Higher scores represented greater pain and poorer function.

We used the 13-item Pain Catastrophizing Scale (PCS) to measure patients’ negative or exaggerated attitudes towards pain with a specific focus on rumination, magnification and helplessness [[19](#_ENREF_19), [32](#_ENREF_32), [33](#_ENREF_33)]. Similar to a prior study, we utilized a cutoff of >16 to represent a high degree of pain catastrophizing [[19](#_ENREF_19)].

Statistical Analysis

We performed both crude and adjusted analyses to examine the association of individual-level and area-level SES with pain, function and pain catastrophizing. Individual-level SES was defined using the three aforementioned categories of educational attainment. Area-level SES index scores were grouped and treated in an ordinal manner in both crude and adjusted analyses and were divided into quartiles with the upper two combined (<55, 56-59 and >59) due to similar distribution of outcomes in bivariate analyses.

We first assessed bivariate associations between our two central SES constructs- individual and area-level SES- and a range of covariates using Chi-squared tests. The goal of the bivariate analyses was to obtain crude associations between exposures and outcomes and to identify potential confounders based on their relationship with both the exposures and the outcomes. These covariates included age (<65, >65), sex, race (Caucasian vs. other), BMI (<25, 25.1-30, 30.1-35, >35), and mental health (MHI-5 <68 or >68). We then investigated the relationships between individual and area-level SES and our outcomes of interest- pain WOMAC <30, 30-55, >55), functional status (WOMAC <30, 30-55, >55), and pain catastrophizing (PCS >16 or less).

Two distinct sets of analyses were performed, separately for individual and area-level SES. In the first set of analyses, we defined the outcomes of interest as functional status, pain, and pain catastrophizing, expressed as continuous variables. We performed a second set of multivariate linear regression analyses that allowed for interpretation at the individual subject level and for adjustment by key covariates. We expressed the principle outcomes as the percentages of subjects with poor WOMAC function (WOMAC Function > 55), high WOMAC pain (WOMAC Pain >55) and high pain catastrophizing score (PCS >16). Regression analyses included patient factors identified a priori (age and BMI) given their statistically significant relationship with both SES and the outcomes of interest. We chose to include sex based on prior studies that demonstrate differences by SES and by our outcomes of interest, although the relationship between sex and SES was not significant in our preliminary analyses. The relationship between SES and mental health (MHI-5) was significant at the individual level and of borderline significance at the area level. We felt that the role of depression specifically as a potential confounder of the relationship between SES and the outcomes of interest was less clear and we therefore conducted multivariate analyses both with and without adjustment by MHI-5. Other variables, including race/ethnicity and smoking status, were not adjusted for in this model because they were not significantly associated with both SES and the outcomes. Separate models were used to assess individual and area-level SES both because of collinearity and to examine their separate effects. For each of these key independent variables, separate models were carried out to examine the three outcomes: low function, high pain and high pain catastrophizing. The adjusted least square means of the principle outcomes (proportion of subjects with poor function, high pain, high catastrophizing) were calculated for each individual and area-level SES group and tests for SES trend were performed. All analyses were performed using SAS 9.3, Cary, NC.

**Results**

There were 316 individuals enrolled in the combined cohort; the mean age was 65.9 (SD 8.7) the median was 65.8, 186 (59%) were female, and 278 (88%) were Caucasian **(Table 1**). The mean BMI was 30.5 (SD 6.3), the median BMI was 29.6, 8 percent were current smokers, 17 percent had less than college education, 21 percent had some college and 62 percent were college graduates. The overall mean MHI-5 score for this cohort was 76.2 (SD 17.2) and the median was 80. There were 239 individuals (76.4%) with MHI-5 scores >68. The MHI-5 was significant across individual-level SES groups (p=0.04) and of borderline significance across area-level SES groups (p=0.06). Using the available 296 street addresses and 20 post offices addresses closest to the designated P.O. Boxes, the median area-level SES index score was 59 (mean 59 (SD 6), range 42-78), higher than the median U.S. population SES index score of 51 [[27](#_ENREF_27)]. The overall mean score for WOMAC pain was 41.0 (SD 18.2), for WOMAC function was 41.8 (SD 17.1) and for pain catastrophizing was 12 (SD 10.7).

Unadjusted bivariate analyses demonstrated associations between lower levels of preoperative pain and several variables including: older age (>65), male sex, lower BMI, and higher MHI-5 score (>68; all p-values <0.01). Similarly, older age (p=0.02), lower BMI (p<0.01), and higher MHI-5 score (p<0.01) were associated with better functional status. Older age (p=0.05) and higher MHI-5 scores (p<0.01) were also associated with lower pain catastrophizing scores.

Bivariate analyses examining the key outcomes as continuous variables demonstrated lower mean WOMAC scores for pain and functional limitation and lower mean pain catastrophizing scores among college graduates compared to individuals with less education (all p-values <0.01) (**Table 2a**). Similarly, at the area SES level, bivariate analyses demonstrated lower mean WOMAC scores for pain and functional limitation and lower mean pain catastrophizing scores among the highest area-level SES groups compared to the lowest (all p-values <0.01) (**Table 2b)**.

We observed associations between higher individual-level SES and less pain, better function and lower pain catastrophizing scores (**Figure 1**). Among those with the highest educational attainment (college graduates), 38% presented with low pain compared with 19% among those with the least education (less than college); 32% with the highest education presented with high function compared with 13% with the least education. In addition, 80% of those with the highest education presented with low pain catastrophizing scores compared with 54% with the least education. Similarly, higher area-level SES was associated with less pain, higher function and lower pain catastrophizing scores (**Figure 2).** Comparing the highest two area-level SES quartiles to the lowest, 41% in the highest presented with low pain (WOMAC ≤30) and 31% with high function (WOMAC <30), compared to 25% with low pain and 15% with high function in the lowest area-level SES quartiles. In the highest SES groups, 79% presented with low pain catastrophizing scores (<16) compared with 68% in the lowest SES groups. Lower BMI was also associated with higher area-level SES (p<0.01). We did not find significant associations between race (Caucasian v. non-Caucasian) and pain and function.

We then conducted a series of unadjusted and adjusted analyses examining the percentage of subjects, stratified by SES, with high pain (WOMAC >55), low function (WOMAC >55) and high pain catastrophizing (PCS>16). In our unadjusted analyses, at both the individual and area-level, we found statistically significant trends between higher SES and lower percentages with high pain, low function and high pain catastrophizing (**Additional File 1, Tables 1 and 2**). Multivariate analyses, adjusted for age, sex, and BMI, showed significant associations between lower individual-level SES and poor function (WOMAC >55), high pain (WOMAC >55) and high pain catastrophizing (PCS >16). We found that 33.8% of subjects (95% CI 22.5-45.2) with less than college education (lowest SES) and 20.5% (95% CI 14.6-26.3) of college graduates (highest SES) presented with high pain (p=0.02), while 36.1% (95% CI 25.2-47.0) with the lowest SES and 17.0% (95% CI 11.4-22.5) with the highest presented with poor function (p<0.01) (**Figure 3**)**.** We also observed significantly higher mean pain catastrophizing scores among patients with less than college level education compared with college graduates (p<0.01). Compared to our unadjusted model, at the individual SES level, we saw a slight attenuation in percentage of those in the lowest SES group with high pain, low function and high PCS, however values were within less than 7% for all SES strata and statistical significance was consistently achieved.

In parallel adjusted analysis that examined area-level SES, we found that 34.6% (95% CI 25.6-43.6) with the lowest area SES and 18.2% (95% CI 11.3-25.1) with the highest presented with high pain (p=0.01), while 31.1% (95% CI 22.4-39.8) with the lowest area SES and 18.2% (95% CI 11.5-24.9) with the highest presented with poor function (p=0.03) (**Figure 4**). We did not observe a statistically significant trend in the relationship between area-level SES and pain catastrophizing scores (p=0.12).

We conducted additional analyses of the associations between education or area-level SES and our three dependent variables, in which we added mental health, as measured by the MHI-5, as a covariate to age, sex and BMI. The adjusted mean percentages of subjects with high pain, low function or high PCS were virtually identical with and without adjustment for MHI5. (**Additional File 1,Tables 1 and 2**).

**Discussion**

Higher socioeconomic status, whether measured at the individual or the area-level, is associated with greater access to discretionary procedures, health behaviors, mental health, and lower morbidity and mortality[[34](#_ENREF_34), [35](#_ENREF_35)]. Studies demonstrate that the association between SES and health spans the entire SES spectrum and is not limited to the poverty threshold [[35](#_ENREF_35)]. In our cohort of individuals with higher area-level SES than the general US population, we demonstrated that individuals from the highest SES areas presented for TKA with less self-reported pain and better functional status than those from lower SES areas. At the individual level, education, a frequently used proxy for SES, provided parallel findings- those with the highest level of education presented with significantly less pain, better function and lower pain catastrophizing scores than those with less education.

A number of factors may have contributed to these findings. Lower SES individuals may wait longer to present for their procedure, which may be related to health system delays, physician bias, patient education or patient preference[[36](#_ENREF_36)]. Barriers such as language, literacy and access to educational resources may also contribute. While prior studies demonstrated racial/ethnic differences in willingness to undergo joint replacement surgery, differences by SES have not been thoroughly examined.

A number of studies also describe a relationship between psychological health and SES[[35](#_ENREF_35), [37](#_ENREF_37)]. Lower individual and area-level SES contributes to increased psychological stress and poor mental health [[38](#_ENREF_38)]. Conversely, prolonged poor psychological health may result in lower SES [[39](#_ENREF_39)]. In our cohort, while lower scores on the mental health index were associated with increased pain and decreased function, the relationship with SES was of borderline significance. We observed little difference in the relationship between SES and pain, function and pain catastrophizing in our models that adjusted for MHI-5 compared to those that did not, suggesting that SES effects are independent of mental health.

Psychological health and pain catastrophizing, a reflection of stress and coping, are associated with outcomes follow joint replacement [[19-22](#_ENREF_19), [40](#_ENREF_40)]. A study in scleroderma patients examined pain catastrophizing as a possible mediator or moderator of the relationship between education and pain [[41](#_ENREF_41)]. The authors found that while catastrophizing and depression contributed significantly to education-related differences in report of pain, lower educational attainment remained a significant risk factor for poor pain-related outcomes. Similarly, in a study of patients with fibromyalgia, lower SES, as measured by education level, exhibited greater pain catastrophizing and more severe disease.[[42](#_ENREF_42)] In our adjusted analyses, we found a significant association between higher educational attainment and lower pain catastrophizing scores. However we did not find a significant relationship between area-level SES and pain catastrophizing. We are not sure why neighborhood characteristics appear to play less of a role than individual-level factors on this aspect of psychological health, and suggest this as an important area for further inquiry.

Among college graduates- the highest individual SES group- we found that 38% presented with low pain and 32% with high function while only 20% presented with high pain, and only 16% with poor function. Similarly, among the highest area-level SES groups, we found that 41% presented with low pain and 31% with high function, while only 15% presented with high pain or with poor function. Our finding, that among patients who presented for TKA, significantly fewer with higher SES had high levels of pain and low levels of function, raises the question of whether TKA was indicated for this population. One prior study examined area the role of area variation in appropriate TKA use and among individuals with severe arthritis, demonstrated a higher percentage willing to undergo joint replacement surgery in geographic areas with higher rates of arthroplasty. These high-rate areas had a similar percent of lower income than low-rate areas, but a lower percentage of high school graduates[[43](#_ENREF_43)]. In the population studied, it did not seem that SES factors played a role on willingness to undergo arthroplasty, although it was not explicitly investigated. Prior studies in fields outside of rheumatology and orthopedics have described potential underuse – necessary procedures not performed- among lower SES individuals [[44](#_ENREF_44)]. However there are few studies to date that examine overuse – inappropriate procedures performed- among higher SES groups [[45](#_ENREF_45), [46](#_ENREF_46)]. In cardiovascular disease, appropriateness and necessity criteria were developed for cardiac revascularization procedures. Notable racial/ethnic and gender differences in procedure use among those for whom it was deemed appropriate, were described [[47](#_ENREF_47), [48](#_ENREF_48)]. In the joint replacement literature, one study showed that when explicit appropriateness criteria based on WOMAC scores for pain, function and stiffness was applied to TKA and total hip arthroplasty surgery patients, those who were deemed to be appropriate candidates experienced the greatest post-operative improvement in health-related quality of life [[49](#_ENREF_49)]. The scope of our study was limited only to WOMAC pain and function scores preoperatively in a cross-sectional population of individuals undergoing TKA. Therefore, we cannot conclude whether the TKAs performed were appropriate or not. However, the small percentage of individuals with high SES with high pain and poor function undergoing TKA, does raise the question of whether other factors specific to this group contribute to easier, and possibly earlier access to the procedure.

Another potential explanation for our findings may be that higher SES individuals undergo TKA surgery at appropriate times while lower SES individuals wait too long for their procedure, which may result in poorer outcomes.[[5-8](#_ENREF_5)] It is plausible that individuals with higher SES may have both better access to orthopedic care and a lower threshold to seek TKA surgery compared to those with lower SES. A slight improvement in quality of life might be seen as readily attainable and therefore desirable. The loss of work during recovery may be less of a factor for higher SES individuals, and caretakers and social support may be more available. One study examined outcome expectations for joint replacement surgery and found that the differences by race were attenuated when employment status, income and education were added to the regression model[[13](#_ENREF_13)]. Certain factors that we observed, such as less smoking and lower BMI among higher SES individuals may also render them better surgical candidates increasing the likelihood that the TKA would be offered. In our cohort, the majority of patients are Medicare recipients and therefore insurance status likely plays less of a role for both the patient and the surgeon.

In addition, our study both complements and extends the findings of two studies in the Johnston County Osteoarthritis cohort that demonstrated significant associations between individual and community SES and radiographic knee osteoarthritis.[[15](#_ENREF_15), [50](#_ENREF_50)] In their subset of patients who described symptoms, less of an association between SES and pain and disability was seen.[[15](#_ENREF_15)] To further explore this, our study focused specifically on the population of individuals who were symptomatic enough to undergo TKA and we found a significant relationship between pain, function and SES. In addition, in this population we extended prior work by assessing the association between SES and pain catastrophizing, an important predictor of TKA outcomes.

Results of this study should be viewed in light of several limitations. First, our cohort is comprised of predominately Caucasian individuals receiving care at one academic, tertiary care medical center. Therefore, the relationship between race/ethnicity and our outcomes of pain, function and pain catastrophizing could not be examined comprehensively and our findings may not be generalizable to other groups. In addition, the area-level SES for our population is higher than the U.S. population median. As a result, our findings are more likely to reflect less pain and better function at presentation for TKA than delays to care among the lower SES groups. In line with this, while we examined both an individual-level measure of SES (educational attainment as a proxy) and a validated area-level composite index that included education, occupation, income and crowding at the smallest area-level available, other aspects that contribute to SES such as discrimination and rank, may not be captured by either measure. At the individual level, we cannot account for differences in quality of higher education, or for the possibility of misreporting. However, our finding of similar results using either the individual or the area-level SES supports the validity of our measures. In addition, while efforts were made to obtain exact addresses, 20 (6%) were post office boxes and therefore the post office addresses were geocoded. It is therefore possible that the area-level SES for these individuals was misclassified. In addition, our objective of this cross-sectional study was to investigate sociodemographic factors that contribute to baseline pain, function and pain catastrophizing at the time of presentation of TKA. While the chosen measures have all been shown to correlate with postoperative outcomes, this evaluation was beyond the scope of our study. Further, we chose to examine pain catastrophizing as a separate outcome of interest in our models given a relationship both with presentation for TKA and with surgical outcomes. It is plausible that pain catastrophizing, as a coping mechanism, may mediate the relationship between SES and pain and function. [[51](#_ENREF_51), [52](#_ENREF_52)]

**Conclusion**

In conclusion, in our cohort, we found that individuals with higher educational attainment and from higher SES areas were significantly less likely to present for TKA with high pain and poor function compared to individuals with low educational attainment and from low SES areas. We found a significant relationship between higher educational attainment and lower pain catastrophizing scores. We also identified an association between older age and better function and lower catastrophizing, which is an important topic to examine further in future research. Overall, this is the first U.S.-based study to specifically examine the association between individual and area-level SES and pain, function, and pain catastrophizing among individuals undergoing TKA. These factors have been shown to be important predictors of TKA outcomes. Additional strengths of this study include the use of both individual and area-level geocoded measures to allow for a multifaceted understanding of the relationship of SES with our outcomes of interest. We also conducted our analyses using a relatively large cohort of individuals with complete, comprehensive demographic information and validated survey measures. Further studies are necessary to confirm a trend of less severe pain and better function at baseline among individuals with higher individual and area-level SES compared to lower. Additional research is also needed to develop and apply appropriateness criteria to examine potential sociodemographic disparities in TKA use and outcomes.

**Abbreviations:** Total knee arthroplasty (TKA), Socioeconomic status (SES), Western Ontario and McMaster Universities Arthritis Index (WOMAC), Pain Catastrophizing Score (PCS)

**Competing interests:**  The authors (CHF, YD, JNK, LDF, EL) have no competing interest to disclose.

**Acknowledgements**

All authors were involved in drafting the manuscript and revising it critically for important intellectual content. This work was supported by NIAMS grants T32 AR055885 (CHF); K24 AR057827 (EL); P60 AR047782 (JNK, YD). CHF is currently supported by the Lupus Foundation of America Career Development Award and receives research support from Pfizer Pharmaceuticals. The funding sources played no role in the design of the study, the collection, analysis and interpretation of data, the writing of the manuscript or the decision to submit the manuscript.

**Author Contributions**

CHF, YD, JNK, LDF, EL contributed to the conception and design of this study. CHF, YD, JNK, LDF, EL participated in the acquisition of data. CHF, YD, JNK, EL participated in the analysis and interpretation of data. All authors read and approved the final manuscript.

**References**

1. Cram P, Lu X, Kates SL, Singh JA, Li Y, Wolf BR: **Total knee arthroplasty volume, utilization, and outcomes among Medicare beneficiaries, 1991-2010**. *JAMA* 2012, **308**(12):1227-1236.

2. Mahomed NN, Barrett JA, Katz JN, Phillips CB, Losina E, Lew RA, Guadagnoli E, Harris WH, Poss R, Baron JA: **Rates and outcomes of primary and revision total hip replacement in the United States medicare population**. *J Bone Joint Surg Am* 2003, **85-A**(1):27-32.

3. Skinner J, Weinstein JN, Sporer SM, Wennberg JE: **Racial, ethnic, and geographic disparities in rates of knee arthroplasty among Medicare patients**. *N Engl J Med* 2003, **349**(14):1350-1359.

4. Dunlop DD, Song J, Manheim LM, Chang RW: **Racial disparities in joint replacement use among older adults**. *Med Care* 2003, **41**(2):288-298.

5. Dixon T, Shaw M, Ebrahim S, Dieppe P: **Trends in hip and knee joint replacement: socioeconomic inequalities and projections of need**. *Ann Rheum Dis* 2004, **63**(7):825-830.

6. Agabiti N, Picciotto S, Cesaroni G, Bisanti L, Forastiere F, Onorati R, Pacelli B, Pandolfi P, Russo A, Spadea T *et al*: **The influence of socioeconomic status on utilization and outcomes of elective total hip replacement: a multicity population-based longitudinal study**. *Int J Qual Health Care* 2007, **19**(1):37-44.

7. Ackerman IN, Graves SE, Wicks IP, Bennell KL, Osborne RH: **Severely compromised quality of life in women and those of lower socioeconomic status waiting for joint replacement surgery**. *Arthritis Rheum* 2005, **53**(5):653-658.

8. Ackerman IN, Ademi Z, Osborne RH, Liew D: **Comparison of health-related quality of life, work status, and health care utilization and costs according to hip and knee joint disease severity: a national Australian study**. *Phys Ther* 2013, **93**(7):889-899.

9. Hawker GA, Wright JG, Glazier RH, Coyte PC, Harvey B, Williams JI, Badley EM: **The effect of education and income on need and willingness to undergo total joint arthroplasty**. *Arthritis Rheum* 2002, **46**(12):3331-3339.

10. Ibrahim SA, Siminoff LA, Burant CJ, Kwoh CK: **Differences in expectations of outcome mediate African American/white patient differences in "willingness" to consider joint replacement**. *Arthritis Rheum* 2002, **46**(9):2429-2435.

11. Ibrahim SA, Siminoff LA, Burant CJ, Kwoh CK: **Understanding ethnic differences in the utilization of joint replacement for osteoarthritis: the role of patient-level factors**. *Med Care* 2002, **40**(1 Suppl):I44-51.

12. Hausmann LR, Mor M, Hanusa BH, Zickmund S, Cohen PZ, Grant R, Kresevic DM, Gordon HS, Ling BS, Kwoh CK *et al*: **The effect of patient race on total joint replacement recommendations and utilization in the orthopedic setting**. *J Gen Intern Med* 2010, **25**(9):982-988.

13. Groeneveld PW, Kwoh CK, Mor MK, Appelt CJ, Geng M, Gutierrez JC, Wessel DS, Ibrahim SA: **Racial differences in expectations of joint replacement surgery outcomes**. *Arthritis Rheum* 2008, **59**(5):730-737.

14. Peters TJ, Sanders C, Dieppe P, Donovan J: **Factors associated with change in pain and disability over time: a community-based prospective observational study of hip and knee osteoarthritis**. *Br J Gen Pract* 2005, **55**(512):205-211.

15. Cleveland RJ, Luong ML, Knight JB, Schoster B, Renner JB, Jordan JM, Callahan LF: **Independent associations of socioeconomic factors with disability and pain in adults with knee osteoarthritis**. *BMC Musculoskelet Disord* 2013, **14**:297.

16. Fortin PR, Penrod JR, Clarke AE, St-Pierre Y, Joseph L, Belisle P, Liang MH, Ferland D, Phillips CB, Mahomed N *et al*: **Timing of total joint replacement affects clinical outcomes among patients with osteoarthritis of the hip or knee**. *Arthritis Rheum* 2002, **46**(12):3327-3330.

17. Desmeules F, Dionne CE, Belzile EL, Bourbonnais R, Champagne F, Fremont P: **Determinants of pain, functional limitations and health-related quality of life six months after total knee arthroplasty: results from a prospective cohort study**. *BMC Sports Sci Med Rehabil* 2013, **5**:2.

18. Fortin PR, Clarke AE, Joseph L, Liang MH, Tanzer M, Ferland D, Phillips C, Partridge AJ, Belisle P, Fossel AH *et al*: **Outcomes of total hip and knee replacement: preoperative functional status predicts outcomes at six months after surgery**. *Arthritis Rheum* 1999, **42**(8):1722-1728.

19. Riddle DL, Wade JB, Jiranek WA, Kong X: **Preoperative pain catastrophizing predicts pain outcome after knee arthroplasty**. *Clin Orthop Relat Res* 2010, **468**(3):798-806.

20. Ayers DC, Franklin PD, Ploutz-Snyder R, Boisvert CB: **Total knee replacement outcome and coexisting physical and emotional illness**. *Clin Orthop Relat Res* 2005, **440**:157-161.

21. Sullivan M, Tanzer M, Stanish W, Fallaha M, Keefe FJ, Simmonds M, Dunbar M: **Psychological determinants of problematic outcomes following Total Knee Arthroplasty**. *Pain* 2009, **143**(1-2):123-129.

22. Sullivan M, Tanzer M, Reardon G, Amirault D, Dunbar M, Stanish W: **The role of presurgical expectancies in predicting pain and function one year following total knee arthroplasty**. *Pain* 2011, **152**(10):2287-2293.

23. Winkleby MA, Jatulis DE, Frank E, Fortmann SP: **Socioeconomic status and health: how education, income, and occupation contribute to risk factors for cardiovascular disease**. *Am J Public Health* 1992, **82**(6):816-820.

24. Krieger N, Chen JT, Waterman PD, Soobader MJ, Subramanian SV, Carson R: **Geocoding and monitoring of US socioeconomic inequalities in mortality and cancer incidence: does the choice of area-based measure and geographic level matter?: the Public Health Disparities Geocoding Project**. *Am J Epidemiol* 2002, **156**(5):471-482.

25. Krieger N, Chen JT, Waterman PD, Rehkopf DH, Subramanian SV: **Race/ethnicity, gender, and monitoring socioeconomic gradients in health: a comparison of area-based socioeconomic measures--the public health disparities geocoding project**. *Am J Public Health* 2003, **93**(10):1655-1671.

26. Kawachi I, Kennedy BP: **Income inequality and health: pathways and mechanisms**. *Health Serv Res* 1999, **34**(1 Pt 2):215-227.

27. **Chapter 3: Creation of New Race-Ethnicity Codes and SES Indicators for Medicare Beneficiaries** [<http://www.ahrq.gov/research/findings/final-reports/medicareindicators/medicareindicators3.html>]

28. Berwick DM, Murphy JM, Goldman PA, Ware JE, Jr., Barsky AJ, Weinstein MC: **Performance of a five-item mental health screening test**. *Med Care* 1991, **29**(2):169-176.

29. Rumpf HJ, Meyer C, Hapke U, John U: **Screening for mental health: validity of the MHI-5 using DSM-IV Axis I psychiatric disorders as gold standard**. *Psychiatry Res* 2001, **105**(3):243-253.

30. Yamazaki S, Fukuhara S, Green J: **Usefulness of five-item and three-item Mental Health Inventories to screen for depressive symptoms in the general population of Japan**. *Health Qual Life Outcomes* 2005, **3**:48.

31. Bellamy N, Buchanan WW, Goldsmith CH, Campbell J, Stitt LW: **Validation study of WOMAC: a health status instrument for measuring clinically important patient relevant outcomes to antirheumatic drug therapy in patients with osteoarthritis of the hip or knee**. *J Rheumatol* 1988, **15**(12):1833-1840.

32. Sullivan MJL BS, Pivik J.: **The Pain Catastrophizing Scale: Development and Validation.** *Psychological Assessment* 1995, **7**:524-532.

33. Kleiman V, Clarke H, Katz J: **Sensitivity to pain traumatization: a higher-order factor underlying pain-related anxiety, pain catastrophizing and anxiety sensitivity among patients scheduled for major surgery**. *Pain Res Manag* 2011, **16**(3):169-177.

34. Gittelsohn AM, Halpern J, Sanchez RL: **Income, race, and surgery in Maryland**. *Am J Public Health* 1991, **81**(11):1435-1441.

35. Adler NE, Boyce T, Chesney MA, Cohen S, Folkman S, Kahn RL, Syme SL: **Socioeconomic status and health. The challenge of the gradient**. *Am Psychol* 1994, **49**(1):15-24.

36. Siciliani L, Verzulli R: **Waiting times and socioeconomic status among elderly Europeans: evidence from SHARE**. *Health Econ* 2009, **18**(11):1295-1306.

37. Gallo LC, Matthews KA: **Understanding the association between socioeconomic status and physical health: do negative emotions play a role?** *Psychol Bull* 2003, **129**(1):10-51.

38. Jones-Rounds ML, Evans GW, Braubach M: **The interactive effects of housing and neighbourhood quality on psychological well-being**. *J Epidemiol Community Health* 2013.

39. Klebanov P, Brooks-Gunn J, Duncan G: **Does Neighborhood and Family Poverty Affect Mother's Parenting, Mental Health and Social Support?** *Journal of Marriage and Family* 1994, **56**(2):441-455.

40. Wise BL, Niu J, Zhang Y, Wang N, Jordan JM, Choy E, Hunter DJ: **Psychological factors and their relation to osteoarthritis pain**. *Osteoarthritis Cartilage* 2010, **18**(7):883-887.

41. Edwards RR, Goble L, Kwan A, Kudel I, McGuire L, Heinberg L, Wigley F, Haythornthwaite J: **Catastrophizing, pain, and social adjustment in scleroderma: relationships with educational level**. *Clin J Pain* 2006, **22**(7):639-646.

42. Fitzcharles MA, Rampakakis E, Ste-Marie PA, Sampalis JS, Shir Y: **The association of socioeconomic status and symptom severity in persons with fibromyalgia**. *J Rheumatol* 2014, **41**(7):1398-1404.

43. Hawker GA, Wright JG, Coyte PC, Williams JI, Harvey B, Glazier R, Wilkins A, Badley EM: **Determining the need for hip and knee arthroplasty: the role of clinical severity and patients' preferences**. *Med Care* 2001, **39**(3):206-216.

44. Philbin EF, McCullough PA, DiSalvo TG, Dec GW, Jenkins PL, Weaver WD: **Socioeconomic status is an important determinant of the use of invasive procedures after acute myocardial infarction in New York State**. *Circulation* 2000, **102**(19 Suppl 3):III107-115.

45. Kahan JP, Bernstein SJ, Leape LL, Hilborne LH, Park RE, Parker L, Kamberg CJ, Brook RH: **Measuring the necessity of medical procedures**. *Med Care* 1994, **32**(4):357-365.

46. Kahan JP, Park RE, Leape LL, Bernstein SJ, Hilborne LH, Parker L, Kamberg CJ, Ballard DJ, Brook RH: **Variations by specialty in physician ratings of the appropriateness and necessity of indications for procedures**. *Med Care* 1996, **34**(6):512-523.

47. Epstein AM, Weissman JS, Schneider EC, Gatsonis C, Leape LL, Piana RN: **Race and gender disparities in rates of cardiac revascularization: do they reflect appropriate use of procedures or problems in quality of care?** *Med Care* 2003, **41**(11):1240-1255.

48. Hannan EL, van Ryn M, Burke J, Stone D, Kumar D, Arani D, Pierce W, Rafii S, Sanborn TA, Sharma S *et al*: **Access to coronary artery bypass surgery by race/ethnicity and gender among patients who are appropriate for surgery**. *Med Care* 1999, **37**(1):68-77.

49. Quintana JM, Escobar A, Arostegui I, Bilbao A, Azkarate J, Goenaga JI, Arenaza JC: **Health-related quality of life and appropriateness of knee or hip joint replacement**. *Arch Intern Med* 2006, **166**(2):220-226.

50. Callahan LF, Cleveland RJ, Shreffler J, Schwartz TA, Schoster B, Randolph R, Renner JB, Jordan JM: **Associations of educational attainment, occupation and community poverty with knee osteoarthritis in the Johnston County (North Carolina) osteoarthritis project**. *Arthritis Res Ther* 2011, **13**(5):R169.

51. Kim HJ, Kim SC, Kang KT, Chang BS, Lee CK, Yeom JS: **Influence of educational attainment on pain intensity and disability in patients with lumbar spinal stenosis: mediation effect of pain catastrophizing**. *Spine (Phila Pa 1976)* 2014, **39**(10):E637-644.

52. Day MA, Thorn BE: **The relationship of demographic and psychosocial variables to pain-related outcomes in a rural chronic pain population**. *Pain* 2010, **151**(2):467-474.

**Table 1.** Baseline characteristics of the overall preoperative TKA cohort, and stratified by area-level socioeconomic status (SES)

| **Characteristics** | **Overall Cohort**  N=316 | **SES 1**  N=85 | **SES 2**  N=87 | **SES 3**  N=144 | **p-value*** |
| --- | --- | --- | --- | --- | --- |
| **Age – mean (SD)** | 65.9 (8.7)  Median: 65.8 | 63.2 (8.0) | 65.6 (9.8) | 67.6 (8.1) | **<0.01** |
| **Sex: Female- n (%)** | 186 (59) | 52 (61) | 50 (57) | 84 (58) | 0.89 |
| **Race: Caucasian – n (%)** | 278 (88) | 65 (76) | 78 (90) | 135 (94) | **<0.01** |
| **BMI – mean (SD)** | 30.5 (6.3)  Median: 29.6 | 32.5 (6.9) | 31.0 (6.5) | 28.9 (5.4) | **<0.01** |
| **Current smokers – n (%)** | 24 (8) | 10 (12) | 2 (2) | 12 (8) | **0.04** |
| **Educational attainment – n (%)**  **Less than College**  **Some college**  **College graduates** | 53 (17)  65 (21)  195 (62) | 22 (26)  20 (24)  43 (51) | 15 (17)  22 (26)  49 (57) | 16 (11)  23 (16)  103 (73) | **0.01** |
| **Mental Health Index Score – mean (SD)** | 76.2 (17.2)  Median: 80 | 73.6 (18.0) | 74.8 (17.8) | 78.5 (16.2) | 0.06 |
| *P-values compare SES groups with Kruskal-Wallis test for continuous and Fisher’s Exact test for categorical variables; SES 1 is the lowest area-level SES quartiles, SES 3 is the highest two quartiles combined. | | | | | |

**Table 2a.** Mean values of the outcomes of pain, function and pain catastrophizing from bivariate analyses for individual SES represented by educational attainment level

| **Dependent Variable** | **Overall Cohort**  N=313** | **Less than College**  N=53 | **Some College**  N=65 | **College Graduates**  N=195 | **p-value*** |
| --- | --- | --- | --- | --- | --- |
| **Pain – mean (SD)** | 41.0 (18.2) | 48.9 (20.1) | 43.1 (20.3) | 38.2 (16.3) | **<0.01** |
| **Functional Status- mean (SD)** | 41.8 (17.1) | 49.6 (17.1) | 44.6 (16.5) | 38.7 (16.6) | **<0.01** |
| **Pain Catastrophizing- mean (SD)** | 12.0 (10.7) | 17.6 (13.9) | 14.3 (10.9) | 9.8 (8.8) | **<0.01** |
| *P-values determined from ANOVA  **3 subjects were missing educational attainment data | | | | | |

**Table 2b.** Mean values of the outcomes of pain, function and pain catastrophizing from bivariate analyses for each area-level SES**^†^** group

| **Dependent Variable** | **Overall Cohort**  N=316 | **SES 1**  N=85 | **SES 2**  N=87 | **SES 3**  N=144 | **p-value*** |
| --- | --- | --- | --- | --- | --- |
| **Pain – mean (SD)** | 41.0 (18.2) | 46.2 (20.2) | 42.4 (18.4) | 37.2 (15.9) | **<0.01** |
| **Functional Status- mean (SD)** | 41.8 (17.1) | 46.6 (17.8) | 42.8 (17.5) | 38.4 (15.8) | **<0.01** |
| **Pain Catastrophizing- mean (SD)** | 12.0 (10.7) | 13.8 (12.1) | 13.3 (11.5) | 10.2 (8.9) | **0.01** |
| *P-values determined from Ward tests of beta coefficients in a model assuming linear trend of SES index groups: **^†^**SES 1 is the lowest quartile, SES 3 is the highest two quartiles combined. | | | | | |

**Figure 1:** Unadjusted percentages of subjects with low pain (WOMAC <30), moderate pain (WOMAC 30-55) and high pain (WOMAC >55), high function (WOMAC <30), moderate function (WOMAC 30-55) and low function (WOMAC >55), and high pain catastrophizing score (PCS >16) and low pain catastrophizing score (PCS <16) by individual-level SES (educational attainment)

**Figure 2.** Unadjusted percentages of subjects with low pain (WOMAC <30), moderate pain (WOMAC 30-55) and high pain (WOMAC >55), high function (WOMAC <30), moderate function (WOMAC 30-55) and low function (WOMAC >55), and high pain catastrophizing score (PCS >16) and low pain catastrophizing score (PCS <16) by area-level SES (SES 3 is highest)

**Figure 3.** Percentage and 95% confidence interval of subjects with high pain or low function (WOMAC >55), or high pain catastrophizing (PCS >16) adjusted for age, sex, and BMI stratified by individual level SES measured by educational attainment

**Figure 4.** Percentage and 95% confidence interval of subjects with high pain or low function (WOMAC >55), or high pain catastrophizing survey scores (PCS > 16) adjusted for age, sex, and BMI stratified by area-level SES

Additional File 1:

Additional File 1, Table 1. Unadjusted and adjusted models indicating the percentage and 95% CI of subjects with high pain (WOMAC >55), low function (WOMAC >55) or high pain catastrophizing (PCS >16) stratified by individual-level SES

Additional File 1, Table 2: Unadjusted and adjusted models indicating the percentage and 95% CI of subjects with high pain (WOMAC >55), low function (WOMAC >55) or high pain catastrophizing (PCS >16) stratified by area-level SES
