# Supplementary material for: Association between socioeconomic status and pain, function and pain catastrophizing at presentation for total knee arthroplasty
Source: BMC Musculoskelet Disord. 2015 Feb 7;16:18. doi: 10.1186/s12891-015-0475-8 (PMC4329215; doi:10.1186/s12891-015-0475-8)
Supplement: Additional file 2: Table S2. — Unadjusted and adjusted models indicating the percentage and 95% CI of subjects with high pain (WOMAC >55), low function (WOMAC >55) or high pain catastrophizing (PCS ≥16) stratified by area-level SES. [file 12891_2015_475_MOESM2_ESM.docx]

**Additional File 1**

**Additional File 1, Table 1: Unadjusted and adjusted models indicating the percentage and 95% CI of subjects with high pain (WOMAC >55), low function (WOMAC >55) or high pain catastrophizing (PCS >16) stratified by individual-level SES**

|  | **Unadjusted Model** | **Model Adjusted by Age, Sex, BMI** | **Model Adjusted by Age, Sex, BMI, MHI-5** |
| --- | --- | --- | --- |
| **High Pain** | | | |
| Less than college  Some college  College graduate  p-value* | 36.5 (24.9-48.2)  29.7 (19.2-40.1)  20.1 (14.1-26.1)  <0.01 | 33.8 (22.5-45.2)  30.1 (20.7-40.9)  20.5 (14.6-26.3)  0.02 | 32.6 (21.3-43.9)  29.9 (19.9-40.0)  21.1 (15.3-26.9)  0.046 |
| **Low Function** | | | |
| Less than college  Some college  College graduate  p-value* | 38.5 (27.4-49.5)  26.6 (16.6-36.6)  16.5 (10.8-22.2)  <0.01 | 36.1 (25.2-47.0)  27.1 (17.4-36.8)  17.0 (11.4-22.5)  <0.01 | 34.2 (23.5-44.8)  25.8 (16.3-35.3)  17.9 (12.5-23.4)  <0.01 |
| **High PCS** | | | |
| Less than college  Some college  College graduate  p-value* | 47.1 (35.1-59.0)  38.1 (27.3-48.9)  20.1 (14.0-26.3)  <0.01 | 46.5 (34.4-58.6)  38.6 (27.8-49.3)  20.2 (14.0-26.3)  <0.01 | 42.9 (31.6-54.2)  36.0 (26.0-46.1)  21.9 (16.1-27.6)  <0.01 |
| p-value* determine by test for trend across education groups | | | |

**Additional File 1, Table 2: Unadjusted and adjusted models indicating the percentage and 95% CI of subjects with high pain (WOMAC >55), low function (WOMAC >55) or high pain catastrophizing (PCS >16) stratified by area-level SES**

|  | **Unadjusted Model** | **Model Adjusted by Age, Sex, BMI** | **Model Adjusted by Age, Sex, BMI, MHI-5** |
| --- | --- | --- | --- |
| **High Pain** | | | |
| SES 1  SES 2  SES 3  p-value* | 38.1 (29.1-47.1)  26.7 (17.8-35.7)  15.6 (8.6-22.6)  <0.01 | 34.6 (25.6-43.6)  25.9 (17.3-34.6)  18.2 (11.3-25.1)  0.01 | 34.2 (25.3-43.1)  25.6 (17.0-34.2)  18.6 (11.8-25.5)  0.01 |
| **Low Function** | | | |
| SES 1  SES 2  SES 3  p-value* | 34.5 (25.8-43.3)  20.9 (12.3-29.6)  15.6 (8.9-22.3)  <0.01 | 31.1 (22.4-39.8)  20.0 (11.6-28.4)  18.2 (11.5-24.9)  0.03 | 30.5 (22.1-39.0)  19.5 (11.3-27.7)  18.9 (12.4-25.4)  0.049 |
| **High PCS** | | | |
| SES 1  SES 2  SES 3  p-value* | 32.5 (23.0-42.1)  35.3 (25.8-44.8)  21.3 (13.9-28.6)  0.04 | 31.2 (21.5-41.0)  34.8 (25.3-44.2)  22.5 (15.0-30.0)  0.12 | 30.0 (21.0-39.1)  33.7 (25.0-42.5)  23.7 (16.8-30.6)  0.22 |
| SES 1 is lowest area-level socioeconomic status, SES 3 is the highest  *p-value determine by test for trend across SES groups | | | |
